# Human adenovirus type 7 infection causes a more severe disease than type 3

**DOI:** 10.1186/s12879-018-3651-2

**Published:** 2019-01-09

**Authors:** Yangxi Fu, Zhengzhen Tang, Zhixu Ye, Shi Mo, Xingui Tian, Ke Ni, Luo Ren, Enmei Liu, Na Zang

**Affiliations:** 1 Department of Respiratory Medicine, Children’s Hospital of Chongqing Medical University, Chongqing, 400014 China; 2 Pediatric Research Institute of Children’s Hospital of Chongqing Medical University, Chongqing Key Laboratory of Pediatrics, Ministry of Education Key Laboratory of Child Development and Disorders, China International Science and Technology Cooperation Base of Child Development and Critical Disorders, Chongqing, 400014 China; 3 State Key Laboratory of Respiratory Disease, National Clinical Research Center for Respiratory Disease, Guangzhou Institute of Respiratory Disease, the First Affiliated Hospital of Guangzhou Medical University, Guangzhou Medical University, Guangzhou, 510180 China; 4 Institute of Biology, Westlake institute for Advanced Study, Hangzhou, 310024 Zhejiang China

**Keywords:** Disease severity, Adenovirus, HAdV-3, HAdV-7

## Abstract

**Background:**

Human adenovirus type 3 (HAdV-3) and 7 (HAdV-7) cause significant morbidity and develop severe complications and long-term pulmonary sequelae in children. However, epidemiologic reports have suggested that nearly all highly severe or fatal adenoviral diseases in children are associated with HAdV-7 rather than HAdV-3. Here, we conduct in-depth investigations to confirm and extend these findings through a comprehensive series of assays in vitro and in vivo as well as clinical correlates.

**Methods:**

A total of 8248 nasopharyngeal aspirate (NPA) samples were collected from hospitalized children with acute respiratory infections in Children’s Hospital of Chongqing Medical University from June 2009 to May 2015. Among 289 samples that tested positive for HAdVs, clinical data of 258 cases of HAdV-3 (127) and HAdV-7 (131) infections were analyzed. All HAdV-positive samples were classified by sequencing the hexon and fiber genes, and compared with clinical data and virological assays. We also performed in vitro assays of virus quantification, viral growth kinetics, competitive fitness, cytotoxicity and C3a assay of the two strains. Mouse adenovirus model was used to evaluate acute inflammatory responses.

**Results:**

Clinical characteristics revealed that HAdV-7 infection caused more severe pneumonia, toxic encephalopathy, respiratory failure, longer mean hospitalization, significantly lower white blood cell (WBC) and platelet counts, compared to those of HAdV-3. In cell culture, HAdV-7 replicated at a higher level than HAdV-3, and viral fitness showed significant differences as well. HAdV-7 also exhibited higher C3a production and cytotoxic effects, and HAdV-7-infected mice showed aggravated pathology and higher pulmonary virus loads, compared to HAdV-3-infected mice. Macrophages in BALF remained markedly high during infection, with concomitant increase in pro-inflammatory cytokines (TNF-α, IL-1β, IFN-γ, and IL-6), compared HAdV-3 infection.

**Conclusions:**

These results document that HAdV-7 replicates more robustly than HAdV-3, and promotes an exacerbated cytokine response, causing a more severe airway inflammation. The findings merit further mechanistic studies that offer the pediatricians an informed decision to proceed with early diagnosis and treatment of HAdV-7 infection.

## Background

Human adenovirus is well-recognized as an important pathogen of respiratory tract infection in childhood [1–3]. More than 70 HAdV serotypes are further subdivided into seven species (A-G) [4, 5]; however, species B (HAdV-3,7,11 and 14), C (HAdV-1,2,5 and 6), and E (HAdV-4) are the ones most associated with respiratory infections [6]. Epidemiological studies have reported an approximately 5–10% HAdV-positive rate among acute respiratory tract infections in children [7, 8]. Adenovirus infections may in fact result in high morbidity and mortality in children, and fatality rates for untreated severe HAdV pneumonia or disseminated disease may exceed 50% [9]. Severe adenovirus infection in children can be complicated with pleural effusions [10], acute respiratory distress syndrome (ARDS) [11], respiratory failure [2], myocarditis [12], and central nervous system dysfunction [13], leading to either mechanical ventilation or extracorporeal life support, even death. Unfortunately, effective adenoviral vaccine for children and specific antiviral drugs against human adenoviruses are currently not available.

HAdV-3 and HAdV-7, belonging to subgenus B1, cause infections that are usually mild and self-limiting in immunocompetent individuals; however, severe, and even life-threatening infections and outbreaks, associated with both type 3 and 7 in children, are increasingly reported [2, 8, 12]. Epidemiology of the disease suggests that HAdV-3 and HAdV-7 are the major types responsible for lower respiratory diseases in children less than 5 years old worldwide [2, 3, 7, 8]. In Beijing, China, HAdV-3, and HAdV-7 were the most common serotypes causing pneumonia from 1958 to 1990 [14]. In Chongqing, China, 53.9% of the HAdV-7-infected patients were diagnosed as having severe pneumonia, significantly higher than the 26.9% of the HAdV-3-infected patients from 2009 to 2014 [3]. In Taiwan, HAdV-3 and HAdV-7, causing complication with respiratory failure, accounted for 53% of infected children; seven cases due to HAdV-7 were among ten deaths reported in a single year, between 2010 and 2011 [2]. In Korea, both HAdV-3 and HAdV-7 cause epidemics of severe lower respiratory tract infections in young children. Overall, 16 and 39%, of the children infected with HAdV-3 and HAdV-7, respectively, required mechanical ventilation [15].

Certain adenovirus serotypes are associated with particular clinical features and severe illness; however, no study has offered persuasive evidence for in the different degrees of disease severity caused by HAdV-3 and HAdV-7 infection because it still limits on the basis of clinical observation. In the present study, therefore, we undertook a comprehensive analysis of the comparative clinical features of HAdV-3 and HAdV-7 infection, as well as a serial of experiments, were performed to better understand the association between severity of the disease and the serotypes of HAdVs.

## Methods

### Participants, demographic data, clinical data analysis

Patients ranging in age from 1 month to 16 years and requiring inpatient treatment due to acute respiratory tract infections (ARTI) at the Department of Respiratory Medicine, Children’s Hospital of Chongqing Medical University between June, 2009 and May, 2015, were enrolled in this study. Parents/guardians provided demographic data and medical history during an interview. Severe disease was defined as respiratory failure confirmed by an abnormal blood gas analysis result (an oxygen saturation level of approximately 90% or less) or toxic encephalopathy. A total of 8248 nasopharyngeal aspirates (NPAs) were collected on the day of hospital admission after obtaining permission from the patients or their guardians. Imaging and laboratory data on admission and during hospitalization were collected. White blood cell (WBC) count > 15,000/μL was defined as leukocytosis, whereas that < 4000/μL was defined as leukopenia.

The study procedure was approved by the ethics committee of the Children’s Hospital of Chongqing Medical University. Informed consent was obtained from parent or guardian of all participants.

### HAdV isolation, identification, and molecular typing

NPAs from HAdV-positive patients were inoculated into HEp-2 cell monolayers and cytopathic effect (CPE) was monitored for 10 days. Viral DNA and RNA were extracted from 200 μL aliquots of the NPA samples by a QIAampMinElute Virus Spin kit (Qiagen, Hilden, Germany). HAdV-positive samples were used to amplify the hexon hypervariable regions (HVRs) 1–6 and fiber genes for subsequent sequencing [16, 17]. The HAdVs strains were molecularly typed by PCR amplification and sequencing of all seven hypervariable regions in the hexon gene, described previously [18].

### HAdV culture and purification

The HAdV-3 (CQ5291) and HAdV-7 strains (CQ4411) used in this study were originally isolated from NPAs of children infected with HAdV. The virus was purified using a standard cesium chloride gradient centrifugation procedure, as described [19]. The concentration of viral particles (VPs) was determined by spectrophotometry according to the A_260_ value. The virus concentration was calculated as VPs/ml = A_260_*dilution*10^12^.

### Virus quantification

Three cell lines (A549, 16HBE, and HEK-293) at 80% confluency in 12-well plates were infected with either HAdV-3 or HAdV-7 at a multiplicity of infection (MOI) of 1 VP/cell. Viruses were cultured as previously described [20, 21]. The culture supernatants and cells were sampled at 48 h post-infection. Viral loads were quantified by quantitative PCR (qPCR). Briefly, 25 μl reaction mixtures were prepared by adding 5 μl of sample nucleic acid extract to 20 μl of iQ Supermix (Bio-Rad, Hercules, CA), primers and probes. The sequences of primers and probes for qPCR and the amplification conditions have been detailed [22, 23]. The real-time HAdV primer sequences were as follows: HAdV-3 forward, 5′- GGGAGACAATATTACTAAAGAAGGTTTGC-3′, HAdV-3 reverse, 5’-CAACTTGAGGCTCTGGCTGATA-3′. The sequence of the HAdV-3 probe was 5’-CACTAC“T”GAAGGAGAAGAAAAGCCCATTTATGCC, which was labeled with 6-carboxyfluorescein (FAM) and internally quenched at “T” with Black Hole Quencher-1 on the 5′- end and phosphate on the 3′-end. The forward primer sequences of HAdV-7 were 5’-GAGGAGCCAG ATATTGATATGGAATT-3′, The reverse primer sequences of HAdV-7 were: 5’-AATTGACATTTTCCGTGTAAAGCA-3′, and probe, 5’-AAGCTGCTGACGCTTTTTCGCCTGA-3′. The HAdV-7 probe was labeled with 6-carboxyfluorescein (FAM) on the 5′-end and 3′-terminally quenched with Black Hole Quencher-1. The thermal cycling condition was as follows: one cycle of 3 min at 95 °C; 45 cycles of 15 s at 95 °C; and 1 min at 60 °C.

### Viral growth kinetics

A549 cells at 80% confluency in 12-well plates were infected with either HAdV-3 or HAdV-7 (MOI = 25 VPs/cell). After 1 h adsorption with rocking every 15 min, the inoculum was removed, 1 ml of fresh DMEM-F12 with 10% FBS was added to each well, and the plates were incubated at 37 °C in 5% CO_2_. The culture media were harvested at 0, 4, 8, 12, 24, 36, 48, 60, 72 and 96 h post-infection. TCID_50_ was used to titer the virus at each time point.

### Viral fitness

A549 cells were infected with a mixture HAdV-3 and HAdV-7 at a ratio of 1:1 and MOI of 10 VPs/cell in standard 12-well cell culture plates, and incubated as described under ‘Viral growth kinetics’. Media supernatants and cells were harvested at 0, 2, 8, 12, 24, 36, 48, 72 h post-infection. Virus copies were quantified at each indicated time point by qPCR described earlier [22, 23].

### Cytotoxicity assay

A549 cells (2.0 × 10^4^) were seeded in each well of 96-well flat bottom plates and incubated at 37 °C in 5% CO_2_ overnight. Thereafter, the media were removed, and cells were infected with HAdV-3 or HAdV-7 (low MOI = 25 VPs/cell, or high MOI = 250 VPs/cell). Infected and uninfected cell viability were assayed at 2, 4, 24, 36, 48 and 60 h post-infection and the absorbance was determined as described [20, 21]. Cell viability was expressed as the ratio, and the mean (mean ± SEM) of 5 replicates of two strains was presented.

### C3a quantification

All samples were collected as described previously, with minor modifications [24]. Purified HAdVs (0.016 g) in PBS were mixed with a 1:10 dilution of normal human serum (NHS) in an assay volume of 20 μl, and incubated for 2, 4 and 10 min at 37 °C. The samples were further diluted 1:500 and tested using an OptEIA human C3a ELISA kit (BD Biosciences), according to the standard procedure provided by the manufacturer.

### Animal model

Female BALB/c mice, 6–8-week old, were purchased from the Animal Laboratory of Chongqing Medical University and housed in a dedicated pathogen-free facility at Chongqing Medical University. The use of all experimental animals strictly met the ethical requirements of the Animal Committee of Chongqing Medical University (license numbers: SYXK(Yu) 2012–0001). The mice under anesthesia were infected intranasally (i.n.) with 5 × 10^9^ VPs/ml of HAdV-3 or HAdV-7 in 100 μl. Mock-infected mice were instilled with 100 μl of phosphate-buffered saline (PBS) under the same conditions. Mice were processed after 3, 5, and 7 days of infection.

### Quantification of pulmonary viral load

For studies of viral load, total viral nucleic acid was directly extracted from the right lung lobe, using a QIAamp mini-viral DNA extraction kit (Qiagen, Hilden, Germany) and following the manufacturer’s recommendations. Quantification of HAdV-3 and HAdV-7 copies were performed as described earlier [22, 23].

### Analysis of inflammatory cells in bronchoalveolar lavage fluid (BALF)

After 3, 5, and 7 days of infection (4 mice in each time point in every group), the animals were euthanatized by intraperitoneal injection of pentobarbital (150 mg/kg). The left pulmonary hilum was closed by clamping, in order to collect BALF from the right side. The specific steps were as described previously [25]. Briefly, bronchoalveolar lavage was performed using 0.25 ml of ice-cold sterile PBS for a total of 6 times. BALF was centrifuged at 2500 rpm for 5 min in cold (4 °C). The supernatant was aliquoted and stored at 80 °C for subsequent cytokine measurements. The precipitate was resuspended in 1 ml of PBS, and the total number of cells was counted under a microscope. The cells were centrifuged, smeared onto slides, fixed, and stained with Diff Quik (Baxter Healthcare Corp, Deerfleld, Miami, FL) to analyze and classify white blood cells. Cell counting was performed using a microscope. A total of 200 random cells was counted from each slide and included macrophages, lymphocytes, and neutrophils.

### Histopathological staining

Mice were sacrificed at 3, 5, and 7 d after infection for pathological analyses. The left lung lobe was collected, fixed in 4% paraformaldehyde for 24 h, dehydrated, and then embedded in paraffin. Lung tissue blocks were cut into 4 μm thick sections that were then stained with hematoxylin and eosin to evaluate lung tissue pathology associated with HAdV-3 and HAdV-7 infection.

### Detection of cytokines

The levels of interferon-γ (IFN-γ), tumor necrosis factor-α (TNF-α), interleukin-1β (IL-1β), and interleukin-6 (IL-6) in BALF were all measured using commercial murine ELISA reagent kits (NeoBioscience, Shenzhen, China) according to the manufacturer’s instructions.

### Statistical analyses

Descriptive statistics were performed for all variables; the continuous variables were summarized as means and standard deviations (SD) or as medians and ranges, and the categorical variables were summarized as frequencies and proportions. The Graph Pad Prism 5.0 software was used for data analysis. Statistical significance was assessed by one- or two-way analysis of variance (ANOVA). One-way ANOVA was used to analyze significant differences between three or more groups. Two-way ANOVA was used to analyze significant differences between two variables. If an overall test was significant, the Tukey’s test was utilized for specific comparisons between individual groups. Differences were considered significant at *P* < 0.05.

## Results

### More severe disease in patients infected with HAdV-7 compared to HAdV-3

Among the 8248 NPAs, 289 specimens were identified as positive for adenovirus, of which HAdV-3 (127) and HAdV-7(131) were the most common types. As shown in Table 1, age and gender distribution, underlying disease, and coinfection were comparable between HAdV-3 and HAdV-7. Co-infection with other respiratory viruses were observed in 57.5 and 40.5% of patients infected with HAdV-3 and HAdV-7, respectively. The most common clinical manifestations of HAdV-3 and HAdV-7 infections, such as cough, wheezing, croup, dry rales, moist rales and fever, including the peak of febrile body temperature (> 39 °C), were not significantly different. In contrast, pneumonia was the most common diagnoses of the patients. Among the 131 HAdV-7-infected patients, 31.3% were diagnosed as having severe pneumonia, significantly higher than that of the HAdV-3-infected patients (31.3% vs 16.5%, *P* = 0.005). When the median duration of hospitalization was analyzed, we found that the HAdV-7 inpatients had a longer mean duration of hospitalization, 8.2 ± 0.6 days (*P* = 0.031). Several severe outcomes, toxic encephalopathy, and respiratory failure were significantly higher in patients with HAdV-7 infection compared with those with HAdV-3 (*P* < 0.05), but no statistically significant differences were found in ICU admission, pleural effusion, suspicious BO and intracranial infection. Notably, one child, infected by HAdV-7, exhibited complications of Acute Respiratory Distress Syndrome (ARDS).

**Table 1:** Demographic and clinical characteristic of children with HAdV-3 and HAdV-7 of admission, 2009-2015

Characteristic	HAdV-3	HAdV-7	*p*
	(*n* = 127)	(*n* = 131)	
Age(years)			
≤ 2 years	91 (71.7)	83 (63.4)	0.155
> 2 years	36 (28.3)	48 (36.6)	
Gender			
Male/female	85/42	90/41	0.761
Underlying diseases	26 (20.5)	24 (18.3)	0.753
Coinfection	73 (57.5)	53 (40.5)	0.851
Symptom, signs			
Cough	118 (92.9)	124 (94.7)	0.974
Fever	89 (70.1)	112 (85.5)	0.656
Peak temperature (> 39)	27 (21.3)	25 (19.1)	0.953
Wheezing	50 (39.4)	89 (32.1)	0.220
Croup	50 (39.4)	42 (32.1)	0.681
Dyspnea	19 (15.0)	31 (23.7)	0.077
Dry rales	57 (44.9)	53 (40.5)	0.627
Moist rales	87 (68.5)	101 (77.1)	0.763
Seizure	1 (0.8)	2 (1.5)	1.000
Diagnosis			
PCF	1 (0.8)	1 (0.8)	1.000
URTI	3 (2.4)	3 (2.3)	1.000
Bronchitis	2 (1.6)	6 (4.6)	0.279
Pneumonia	121 (95.3)	121 (92.4)	0.958
Complication			
Pleural effusion	2 (1.6)	6 (4.6)	0.279
Severe pneumonia	21 (16.5)	41 (31.3)	0.005^a^
Respiratory failure	19 (15.0)	36 (27.5)	0.015^a^
ARDS	0	1 (0.8)	1.000
Suspicious BO	1 (0.8)	2 (1.5)	1.000
Intracranial infection	1 (0.8)	4 (3.1)	0.369
Toxic encephalopathy	1 (0.8)	25 (19.1)	0.0001^a^
Outcomes			
Hospitalization days	7.7 ± 0.7	8.2 ± 0.6	0.031^a^
ICU admission	2 (1.6)	6 (4.6)	0.279

Note: Underlining diseases include premature birth, congenital heart disease, asthma, eczema and tuberculosis

Coinfection with influenza viruses, RSV, PIV, MPV, HRV, COV and HBoV

The numbers in brackets represent the percentage of positive cases

Bold Abbreviations: *PCF* pharyngo conjunctival fever, *URTI* upper respiratory tract infection, *ICU* intensive care unit, *BO* Bronchiolitis obliterans, not determined, *ARDS* acute respiratory distress syndrome

*P*-values indicate comparisons with significance differences (*P* ≤ 0.05)

“^a^”: a significant difference between two groups

Laboratory findings for the HAdV-7-positive inpatients were also significantly different from those infected by HAdV-3 (Table 2). Specifically, the HAdV-7-positive inpatients had lower white blood cell count (10.28 ± 0.51 vs. 11.69 ± 0.46 × 10^9^ cells/L; *P* = 0.041), platelet count (259.9 ± 11.38 vs. 370.2 ± 14.22 × 10^9^ cells/L; *P* = 0.0001). In contrast, hemoglobin and C-reactive protein levels, and the percentages of lymphocytes, neutrophils and positive sputum culture were found to be statistically similar.

**Table 2:** Imaging and laboratory findings of the hospitalized children with HAdV-3 and HAdV-7 infection, 2009–2015

Laboratory Data	HAdV-3	HAdV-7	*p*
	(*n* = 127)	(*n* = 131)	
Radiographic change			
Increased lung texture	13 (10.2)	14 (10.7)	0.992
Alveolar infiltrate	49 (38.6)	61 (46.6)	0.369
Interstitial inflammation	8 (6.3)	8 (6.1)	0.997
Consolidation	9 (7.1)	18 (13.7)	0.894
Atelectasis	2 (1.6)	4 (3.1)	0.683
Pleural effusion	2 (1.6)	6 (4.6)	0.279
Pneumothorax	0	2 (1.5)	1.000
Blood test			
WBC(10^9^cells/L)	11.69 ± 0.46	10.28 ± 0.51	0.041^a^
Leukopenia (< 4000/μL)	2 (1.6)	11 (8.4)	0.904
Neutrophils (%)	50.26 (10–90)	52.32 (14–87)	0.351
Lymphocyte (%)	44.67 (6–90)	43.88 (10–81)	0.794
Platelets(10^9^cells/L)	370.2 ± 14.22	259.9 ± 11.38	0.0001^a^
Hemoglobin(g/L)	111.6 ± 1.12	111.2 ± 1.08	0.779
CRP(mg/L)	10.6 ± 1.14	11.9 ± 1.30	0.499
Positive sputum culture	45 (35.4)	59 (45)	0.420
Positive blood culture	1 (0.8)	0	1.000

Note: Data are shown as medians (interquartile range) or number (%)

Abbreviations: *WBC* white blood cell, *CRP* C-reactive protein

“^a^”: a significant difference between two groups

In the chest radiographs (Table 2), alveolar infiltration, consolidation and pleural effusion were more frequently observed with the HAdV-7 patients compared to HAdV-3 patients but they did not differ significantly. Increased lung texture and interstitial inflammation showed a similar proportion. Two patients infected with type 3, and four patients infected with type 7, had atelectasis. Notably, two patients infected with adenovirus type 7 had complications with pneumothorax.

### Difference in propagation and competitive fitness between HAdV-7 and HAdV-3

**Fig. 1:**
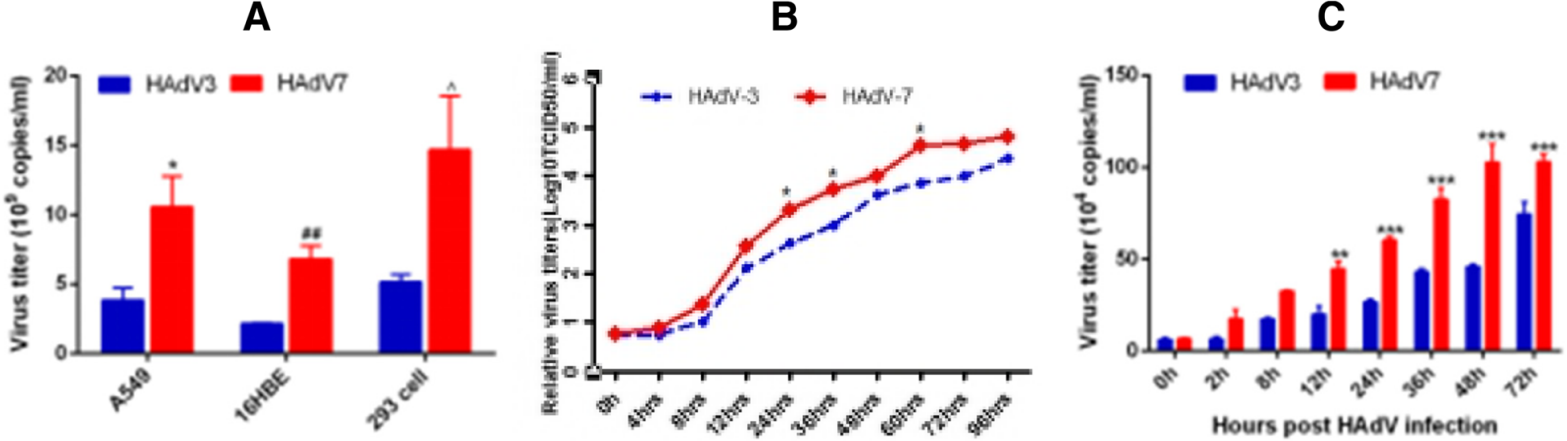
The differences of viral replication post HAdV-3, HAdV-7 infection. **a**. Viral loads of HAdV-3 and HAdV-7 were evaluated in three cell lines at 48 h post-infection; *n* = 4, *P < 0.05, HAdV-7 compared with HAd-V3 in A549; ## *P* < 0.01, HAdV-7 compared with HAdV-3 in 16HBE; ^ *P* < 0.05, HAdV-7 compared with HAdV-3 in 293 cells. **b** Growth kinetics of HAdV-7 or HAdV-3 at 0, 4, 8, 12, 24, 36, 48, 72 and 96 h post-infection in A549 cells; *n* = 3, *, *P* < 0.05, compared with HAdV-3. **c** Competitive replication between HAdV-3 and HAdV-7 in A549 cells (3 replicates each experiment, mean ± SEM); *, **, *** *P* < 0.05, 0.01, 0.001, comparing with HAdV-3

Since type-specific adenovirus infection is known to cause different tissue tropisms and clinical manifestations as indicated before, viral loads and fitness of HAdV-3 and HAdV-7 were evaluated in several human epithelial cells to determine if there were differences. As shown (Fig. 1a), at 48 h post-infection, HAdV-7 replicated to a higher number of viral copies than HAdV-3 in all three cell lines tested, namely, A549, HEK-293 and 16HBE cells. HAdV-7 also exhibited stronger growth than HAdV-3 in A549 cells at 24, 36 and 60 h post-infection (Fig. 1b). In comparing the competitive fitness between HAdV-3 and HAdV-7, however, no significant difference was observed. Lastly, HAdV-7 loads were significantly higher than those of HAdV-3 at 12, 24, 36 and 72 h post-infection (*P* < 0.05) (Fig. 1c).

### Higher cytotoxicity caused by HAdV-7

**Fig. 2:**
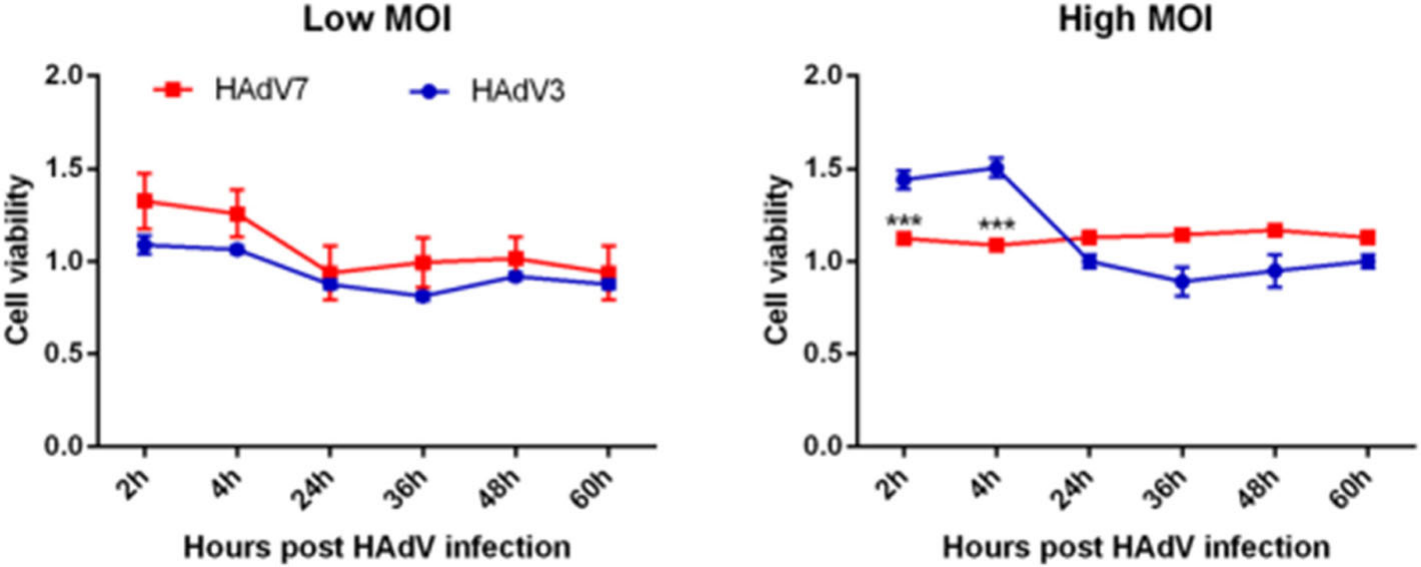
Cytotoxicity of the virus strains, measured by CCK-8 assay. Left: Changes in cell viability for low viral concentration at 2, 4, 24, 36, 48 and 60 h after virus addition. Right = Changes in cell viability (mean ± SEM) for high viral concentration at the same time points. *n* = 5, *** *P* < 0.001, comparing with HAdV-3

Cytotoxicity assay may serve as a measure of cellular distress induced by viral infection. As shown (Fig. 2), at a low MOI of 25 VPs/cell, viability of HAdV-7-infected cells was slightly higher than that of the HAdV-3-infected cells, but there was no statistically significant difference. In contrast, at high MOI of 250 VPs/cell, HAdV-7 induced a remarkably greater decrease in cell viability at both 2 and 4 h post-infection (26.5 and 42.6%, respectively; *P* < 0.0001) (Fig. 2).

### HAdV-7 stimulation results in higher levels of C3a product

**Fig. 3:**
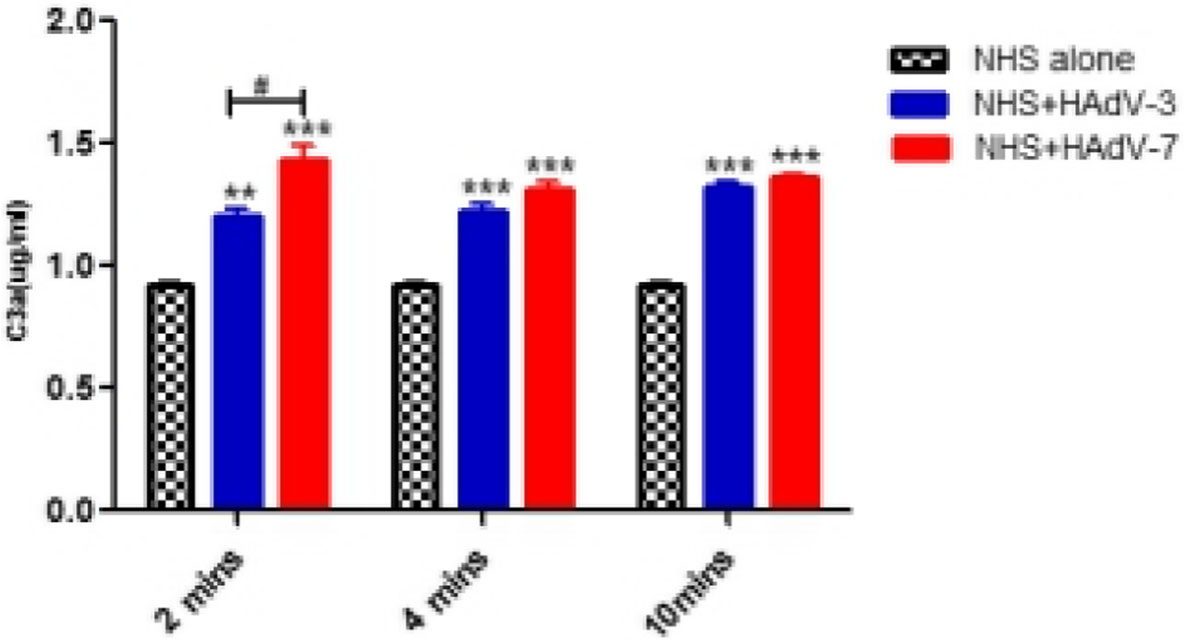
Activation of complement C3a by HAdVs. Normal human serum (NHS) (1:20 dilution) was incubated at 37 °C alone or with purified HAdVs for 2, 4, or 10 min. Samples were analyzed by ELISA for levels of C3a. n = 3, **, *P* < 0.01, ***, *P* < 0.001, NHS + HAdVs infection group compared with NHS alone group. #, *P* < 0.05, NHS + HAdV-7 group compared with NHS + HAdV-3 group

To compare complement activations by HAdV-3 and HAdV-7, the anaphylatoxin C3a, a split fragment of C3 (the step where classical, lectin, and alternative pathways of complement activation merge), was measured in vitro. As shown (Fig. 3), HAdV incubation with serum, compared with NHS incubation alone, resulted in a marked increase of C3a at 2, 4 and 10 mins. Importantly, elevation of C3a by HAdV-7, compared with HAdV-3, was statistically higher at 2 mins (*p* < 0.05).

### Higher viral load and more severe airway inflammation in mice post HAdV-7 infection, compared to HAdV-3 infection

**Fig. 4:**
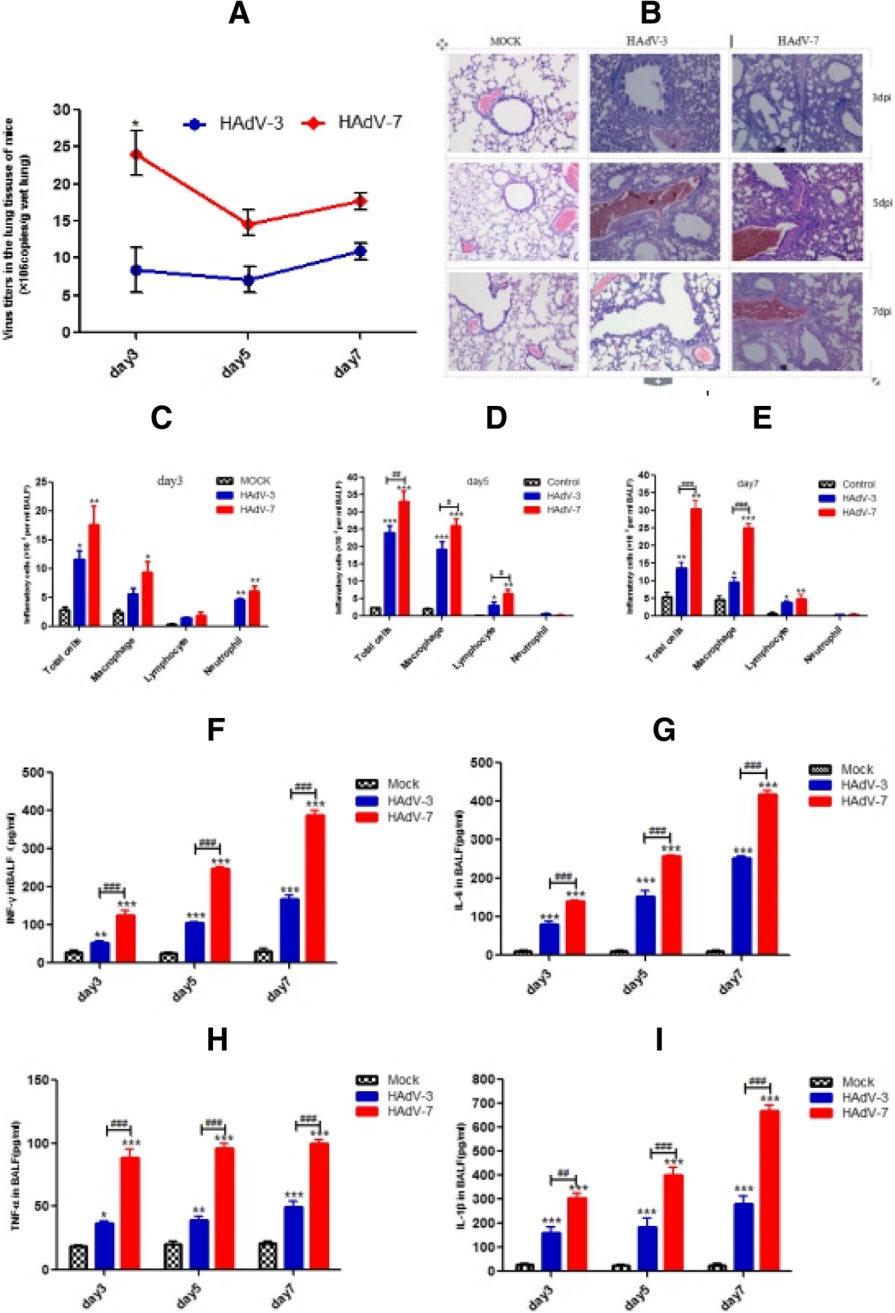
Difference of severity of pathology in mouse infected with HAdV-3 and HAdV-7. The viral load in lung homogenates, the inflammation and the number of infiltrated inflammatory cells in BALF at days 3, 5 and 7 post-infection are shown. **a** Viral load in the lung. **b** H&E staining of lung tissues (magnification 100x); **c**-**e** The number of total cells and the differential counting of cells in BALF. **f**-**i** Concentration of cytokine IL-1β, TNF-α, and IL-6 in BALF; *n* = 4; *,**,*** indicate *P* < 0.05, 0.01, and 0.001, respectively, compared with the mock group; #, ##, ### indicate *P* < 0.05, 0.01, and 0.001, respectively, compared with HAdV-3 group

To better understand the differences in adenovirus-induced airway inflammation, we established a mouse respiratory infection model for human adenovirus. In this model, assays of viral load in the lung tissues revealed that virus copies of HAdV-7-infected groups were higher than those of HAdV-3-infected groups at 3, 5 and 7 days of infection, particularly on day 3, when virus copies in the HAdV-7 group were 1.5-fold higher than the HAdV-3 group (*P* < 0.05) (Fig. 4a). Pulmonary inflammation in mice was also observed following HAdV-3 and HAdV-7 infection at each time point and found to gradually increase from day 3 to day 5, decreasing on day7. Compared with HAdV-3-infected mice, lung tissue damage of HAdV-7-infected mice was also higher on all days of infection (Fig. 4b). Consistent with the morphological changes, the total number of cells in BALF was higher, compared with those in the control group on all days (*P* < 0.001) (Fig. 4c-e); importantly, the HAdV-7 group showed a significantly greater increase in these cells, in comparison with the HAdV-3 group (*p* < 0.05) (Fig. 4d-e). Specifically, abundant macrophages and smaller number of lymphocytes, neutrophils infiltrated into the BALF at all days post-infection. Whereas the neutrophils registered a slight rise, only on day 3, followed by dramatic decrease, the lymphocyte and macrophage numbers remained persistently elevated through days 5 and 7. In addition, macrophages in BALF of the HAdV-7 group were significantly higher than the HAdV-3 group after 5 and 7 days of infection (Fig. 4d-e), while the lymphocytes exhibited significant difference only on day 5 (*P* < 0.05) (Fig. 4d). Neutrophils in BALF of the HAdV-7 group was not significantly different compared with that of the HAdV-3 group at all time points (Fig. 4c-e). Together, these results showed that HAdV-7 infection induced a relatively more severe acute airway inflammation.

### HAdV-7 infection induces higher levels pro–inflammatory cytokines in BALF

The levels of inflammatory mediators associated with airway inflammation, including IL-1β, TNF-α, IFN-γ, and IL-6, were also clearly elevated in BALF at 3, 5, and 7 days post HAdV-3 and HAdV-7 infection. Nevertheless, the levels of IL-1β, IL-6, and TNF-α in the HAdV-7 group were higher than the HAdV-3 group post 3, 5, and 7 days of infection (*p* < 0.01) (Fig. 4f-i).

## Discussion

HAdV-3 and HAdV-7 have been prevalent throughout the world for decades, and both serotypes frequently play an important role in the etiology of lower respiratory infection in children. In the present study, coinfection with HAdV-3 and HAdV-7 with other respiratory viruses accounted for half of the pediatric cases, but coinfection per se did not result in a difference in clinical manifestation or severe clinical outcomes. These results and the severe respiratory diseases and complications, which we identified with HAdV-7 infection in hospitalized children, were all consistent with previous reports [2, 3, 11, 15]. Remarkably, we also found a higher rate (19.1%) of toxic encephalopathy associated with HAdV-7 infection. Previous studies in Japan showed that mild encephalitis/encephalopathy with a reversible splenial lesion (MERS) could be triggered by adenovirus infection [26, 27], which was proposed to be caused by neurotoxins, likely acting as an antigen that induced a strong immune response, leading to a series of symptoms. Recently, silkworm chrysalis ingestion was shown to induce toxic encephalopathy [28]; however, our results constitute the first reported case of toxic encephalopathy associated with adenoviral infection. Earlier studies reported that several viruses, including adenoviruses, influenza A virus, parainfluenza virus, and Japanese encephalitis virus, can use the olfactory nerve as a shortcut into the central nervous system, causing infection of nerve cells and induction of immune response [29]; however, the exact mechanisms of such neurotropic infections remain to be elucidated.

In the current study, we have compared and correlated the biological characteristics of HAdV-3 and HAdV-7 with their properties ex vivo and in vitro. First of all, the results of cellular infectivity (Fig. 1a) indicated that A549, 16HBE, and HEK-293 are susceptible cell lines for the growth of adenovirus ex vivo. Furthermore, a difference of viral loads recorded in A549 and 16HBE cell lines between adenovirus 3 and 7 indicated differential inflammatory reactions were produced from infection of host respiratory epithelial cells by HAdV stimulated the innate immune system that might partially explain the role of HAdV-7 infection in the pathogenesis of a more severe pneumonia. This is likely because the airway epithelial cells not only serve as a passive barrier to infectious particles but also actively participate in the innate immune response to foreign antigens. The immune response of epithelia to infection and antigen exposure involves the release of chemokines and cytokines into the submucosa, which initiates an inflammatory reaction [30]. The results of co-infection of the two viruses indicated that distinctive fitness is type-specific and not interaction. The result of cell viability also provided useful information that was important for understanding type-specific viral virulence (Fig. 2). Taken together, we conclude that the differences observed in the replication, fitness, and virulence of the two adenoviruses are dependent on virus type.

The complement system plays important roles in innate immunity and is vital in the protection against invading pathogens. The complement cascade can be initiated through three main pathways: classical, lectin, and alternative. The classical pathway is mainly antibody-dependent. It is activated by interaction of C1q (a subunit of the first complement protein C1) with the Fc portion of the IgM or IgG immune complex, although activation can also be achieved in an antibody-independent manner by some membrane components of viruses, bacteria and fungi. The lectin pathway is first activated when the mannose-binding lectin, a structural homologue of C1q, binds to carbohydrate moieties on the surface of pathogens, including yeast, bacteria and viruses. The alternative pathway is antibody-independent; it is spontaneously activated on biological surfaces, as well as in plasma and other body fluids, when the level of C3 hydrolysis remains consistently low. This spontaneous cleavage readily initiates amplification of the activation cascades [31]. Although every pathway is triggered independently, all of the complement cascades culminate in the central cleavage of C3 and the generation of its active fragments C3a and C3b, which can bind covalently to viral components to aid in opsonization and phagocytosis. Furthermore, complement activation also mediates the inflammatory reaction via the generation of anaphylatoxins (C3a and C5a) and recruitment of inflammatory cells to the site of infection [32, 33]. Therefore, activation of the complement system leads to inflammation, opsonization, and membrane perturbation.

Previous studies by Johnson et al. and others [24, 34–36] have shown that the complement is strongly activated by a wide range of negative-strand RNA viruses, including parainfluenza virus 5 (PIV5), Nipah virus (NiV), mumps virus (MuV), vesicular stomatitis virus (VSV), and Newcastle disease virus (NDV). For each of these viruses, C3 played an essential role in effective in vitro neutralization by normal human serum (NHS). Here, we explored human adenovirus-induced complement activation to test whether there are differences in infectious HAdV-3 and HAdV-7 in vitro with normal human serum (that contain anti-adenovirus antibodies). As our results demonstrate, higher C3a levels, observed in HAdV-7 infection, rapidly activated human complement within 2 min. Thus, our results also implicate the plasma level of C3a as a marker of the early innate response to adenovirus [37].

In previous studies, our group successfully established a HAdV-7 pneumonia model in the laboratory mouse, and studies in this in vivo model suggested that HMGB1 is a mediator of HAdV-7-induced pulmonary inflammation [38]. Our current study has addressed whether different types of human adenovirus infection result in distinct inflammatory reactions in vivo. In this study, we clearly observed robust virus growth, and also documented significantly higher viral load in HAdV-7-infected mice at 3 days post-infection. These results are different from those reported by Kajon et al. in which only low levels of HAdV-3 and HAdV-7 replication were seen at the early stage of infection in mice [39]. We speculate that the apparent discrepancy is due to the effect of different experimental animal models and detection methods used. Adenovirus infection is characterized pathologically by a time-dependent progression in the type of inflammatory cells present. The initial inflammation is a neutrophilic interstitial infiltration with neutrophilic alveolitis. Subsequently, monocytes become evident and, finally, predominantly lymphocytic infiltrates appear. During the acute stage of HAdV-3 and HAdV-7 infection, we also found that the total number and classes of cells in BALF increased, primarily accounted for by macrophages. Neutrophils only transiently increased at 3 days post-infection. A mixture of mononuclear cells (macrophages and lymphocytes) exclusively became evident at 5 and 7 days post-infection. Macrophages were most prominent than neutrophils and lymphocytes at all time points. These results are fully consistent with several studies, including those of Kajon et al., which showed that HAdV-3 and HAdV-7-induced pneumonia mainly produces neutrophil infiltration at 1 and 2 days post-infection, and affects macrophages [39]. Apart from phagocytosing and killing the pathogens, macrophages also secrete chemokines to recruit cells to the sites of infection. Yoon et al. found that adenovirus type 7 produced a more robust IL-6 and IL-8 response than adenovirus 3 in human bronchial epithelial cells [1, 30]. Diaz et al. compared cytokine responses between type 7 and type 3, and documented significantly higher production of interferon-γ from type 7 [40]. Similarly, subsequent production of IL-1β, induced by macrophages, also reached the highest levels after 3, 5, and 7 days of HAdV-3 and HAdV-7 infection. Asgari et al. have reported that engagement of the C3a receptor triggers IL-1β processing and release via caspase-1 activation [41]. The results are consistent with the immune response of the epithelia to adenovirus infection and antigen exposure, which involves the release of chemokines and cytokines into the submucosa and initiates an inflammatory reaction. Lastly, complement activation mediates the inflammatory reaction via anaphylatoxins (C3a and C5a) and recruitment of inflammatory cells to the site of infection. The inflammation then leads to the recruitment of phagocytes that help clear the invaders. Collectively, we conclude that these innate immune responses play an important role in the initial defense against adenovirus infection.

Although our results implicate a novel type-specific relationship between adenoviral infection, it has potential limitations. The data presented herein only interrogated virological differences; however, adenovirus infection is a very complex process that involves not only the structural proteins of the virus, such as fiber, hexon, and penton, but also includes host and environmental factors. Nonetheless, our data confirm that severe adenovirus infection was associated with HAdV-7 rather than HAdV-3. The significance of the study lies in that the pediatrician should be aware of the importance of early diagnosis and treatment of HAdV-7 infection in children in clinical practice. The findings should also stimulate further studies of mechanisms of the different pathogenicity among human adenovirus serotypes.

## Conclusions

The severity of adenoviral infection, as studied in Chongqing, China, may be correlated to human adenovirus type 7 (HAdV-7) instead of type 3 (HAdV-3). Overall, strain of the HAdV-7 type caused a more severe pneumonia and an exacerbated cytokine response, which also paralleled their more robust replication in cell culture, as compared to HAdV-3. While the exact mechanism of the type-specific pathogenicity merits further investigation, these findings may eventually contribute to better control and treatment of adenoviral infection.

## Abbreviations

BALF: Bronchoalveolar lavage fluid
HAdV-3: Human adenovirus type 3
HAdV-7: Human adenovirus type 7
IFN-γ: Interferon-γ
IL-1β: Interleukin-1β
IL-6: Interleukin-6
MOI: Multiplicity of infection
NHS: Normal human serum
NPA: Nasopharyngeal aspirate
qPCR: Quantitative PCR
TNF-α: Tumor necrosis factor-α
VP: Viral particles

## References

[1] Sun J, Xiao Y, Zhang M, Ao T, Lang S, Wang J (2018). Serum inflammatory markers in patients with adenovirus respiratory infection. Med Sci Monit.

[2] Lai CY, Lee CJ, Lu CY, Lee PI, Shao PL, Wu ET, Wang CC, Tan BF, Chang HY, Hsia SH (2013). Adenovirus serotype 3 and 7 infection with acute respiratory failure in children in Taiwan, 2010-2011. PLoS One.

[3] Wo Y, Lu QB, Huang DD, Li XK, Guo CT, Wang HY, Zhang XA, Liu W, Cao WC (2015). Epidemical features of HAdV-3 and HAdV-7 in pediatric pneumonia in Chongqing, China. Arch Virol.

[4] Hage E, Gerd Liebert U, Bergs S, Ganzenmueller T, Heim A (2015). Human mastadenovirus type 70: a novel, multiple recombinant species D mastadenovirus isolated from diarrhoeal faeces of a haematopoietic stem cell transplantation recipient. J Gen Virol.

[5] Espinola EE, Barrios JC, Russomando G, Mirazo S, Arbiza J (2017). Computational analysis of a species D human adenovirus provides evidence of a novel virus. J Gen Virol.

[6] Scott MK, Chommanard C, Lu X, Appelgate D, Grenz L, Schneider E, Gerber SI, Erdman DD, Thomas A (2016). Human adenovirus associated with severe respiratory infection, Oregon, USA, 2013-2014. Emerg Infect Dis.

[7] Jin Y, Zhang RF, Xie ZP, Yan KL, Gao HC, Song JR, Yuan XH, Hou YD, Duan ZJ (2013). Prevalence of adenovirus in children with acute respiratory tract infection in Lanzhou, China. Virol J.

[8] Lee J, Choi EH, Lee HJ (2010). Comprehensive serotyping and epidemiology of human adenovirus isolated from the respiratory tract of Korean children over 17 consecutive years (1991-2007). J Med Virol.

[9] Lynch JP, Kajon AE (2016). Adenovirus: epidemiology, global spread of novel serotypes, and advances in treatment and prevention. Semin Respir Crit Care Med.

[10] Cho CT, Hiatt WO, Behbehani AM (1973). Pneumonia and massive pleural effusion associated with adenovirus type 7. Am J Dis Child.

[11] Hung KH, Lin LH (2015). Adenovirus pneumonia complicated with acute respiratory distress syndrome: a case report. Medicine.

[12] Treacy A, Carr MJ, Dunford L, Palacios G, Cannon GA, O'Grady A, Moran J, Hassan J, Loy A, Connell J (2010). First report of sudden death due to myocarditis caused by adenovirus serotype 3. J Clin Microbiol.

[13] Huang YC, Huang SL, Chen SP, Huang YL, Huang CG, Tsao KC, Lin TY (2013). Adenovirus infection associated with central nervous system dysfunction in children. J Clin Virol.

[14] Li QG, Zheng QJ, Liu YH, Wadell G (1996). Molecular epidemiology of adenovirus types 3 and 7 isolated from children with pneumonia in Beijing. J Med Virol.

[15] Hong JY, Lee HJ, Piedra PA, Choi EH, Park KH, Koh YY, Kim WS (2001). Lower respiratory tract infections due to adenovirus in hospitalized Korean children: epidemiology, clinical features, and prognosis. Clin Infect Dis.

[16] Biere B, Schweiger B (2010). Human adenoviruses in respiratory infections: sequencing of the hexon hypervariable region reveals high sequence variability. J Clin Virol.

[17] Madisch I, Harste G, Pommer H, Heim A (2005). Phylogenetic analysis of the main neutralization and hemagglutination determinants of all human adenovirus prototypes as a basis for molecular classification and taxonomy. J Virol.

[18] Han G, Niu H, Zhao S, Zhu B, Wang C, Liu Y, Zhang M, Yang S, Liu F, Wan C (2013). Identification and typing of respiratory adenoviruses in Guangzhou, southern China using a rapid and simple method. Virol Sin.

[19] Kanegae Y, Makimura M, Saito I (1994). A simple and efficient method for purification of infectious recombinant adenovirus. Jpn J Med Sci Biol.

[20] Anderson BD, Barr KL, Heil GL, Friary JA, Gray GC (2012). A comparison of viral fitness and virulence between emergent adenovirus 14p1 and prototype adenovirus 14p strains. J Clin Virol.

[21] Liu J, Nian QG, Zhang Y, Xu LJ, Hu Y, Li J, Deng YQ, Zhu SY, Wu XY, Qin ED (2014). In vitro characterization of human adenovirus type 55 in comparison with its parental adenoviruses, types 11 and 14. PLoS One.

[22] Heim A, Ebnet C, Harste G, Pring-Akerblom P (2003). Rapid and quantitative detection of human adenovirus DNA by real-time PCR. J Med Virol.

[23] Lu X, Trujillo-Lopez E, Lott L, Erdman DD (2013). Quantitative real-time PCR assay panel for detection and type-specific identification of epidemic respiratory human adenoviruses. J Clin Microbiol.

[24] Johnson JB, Borisevich V, Rockx B, Parks GD (2015). A novel factor I activity in Nipah virus inhibits human complement pathways through cleavage of C3b. J Virol.

[25] Long X, Li S, Xie J, Li W, Zang N, Ren L, Deng Y, Xie X, Wang L, Fu Z (2015). MMP-12-mediated by SARM-TRIF signaling pathway contributes to IFN-gamma-independent airway inflammation and AHR post RSV infection in nude mice. Respir Res.

[26] Hoshino A, Saitoh M, Oka A, Okumura A, Kubota M, Saito Y, Takanashi J, Hirose S, Yamagata T, Yamanouchi H (2012). Epidemiology of acute encephalopathy in Japan, with emphasis on the association of viruses and syndromes. Brain Dev.

[27] Hibino M, Horiuchi S, Okubo Y, Kakutani T, Ohe M, Kondo T (2014). Transient hemiparesis and hemianesthesia in an atypical case of adult-onset clinically mild encephalitis/ encephalopathy with a reversible splenial lesion associated with adenovirus infection. Intern Med.

[28] Hu H, Wang X, Lv J, Sun J, Xing J, Liu X (2016). The effect of hemoperfusion on patients with toxic encephalopathy induced by silkworm chrysalis ingestion. Scott Med J.

[29] van Riel D, Verdijk R, Kuiken T (2015). The olfactory nerve: a shortcut for influenza and other viral diseases into the central nervous system. J Pathol.

[30] Yoon JS, Kim HH, Lee Y, Lee JS (2007). Cytokine induction by respiratory syncytial virus and adenovirus in bronchial epithelial cells. Pediatr Pulmonol.

[31] Gasque P (2004). Complement: a unique innate immune sensor for danger signals. Mol Immunol.

[32] Walport MJ (2001). Complement. First of two parts. N Engl J Med.

[33] Walport MJ (2001). Complement. Second of two parts. N Engl J Med.

[34] Johnson JB, Lyles DS, Alexander-Miller MA, Parks GD (2012). Virion-associated complement regulator CD55 is more potent than CD46 in mediating resistance of mumps virus and vesicular stomatitis virus to neutralization. J Virol.

[35] Biswas M, Johnson JB, Kumar SR, Parks GD, Elankumarana S (2012). Incorporation of host complement regulatory proteins into Newcastle disease virus enhances complement evasion. J Virol.

[36] Johnson JB, Capraro GA, Parks GD (2008). Differential mechanisms of complement-mediated neutralization of the closely related paramyxoviruses simian virus 5 and mumps virus. Virology.

[37] Tian J, Xu Z, Smith JS, Hofherr SE, Barry MA, Byrnes AP (2009). Adenovirus activates complement by distinctly different mechanisms in vitro and in vivo: indirect complement activation by virions in vivo. J Virol.

[38] Tang Z, Zang N, Fu Y, Ye Z, Chen S, Mo S, Ren L, Liu E (2018). HMGB1 mediates HAdV-7 infection-induced pulmonary inflammation in mice. Biochem Biophys Res Commun.

[39] Kajon AE, Gigliotti AP, Harrod KS (2003). Acute inflammatory response and remodeling of airway epithelium after subspecies B1 human adenovirus infection of the mouse lower respiratory tract. J Med Virol.

[40] Diaz PV, Calhoun WJ, Hinton KL, Avendano LF, Gaggero A, Simon V, Arredondo SM, Pinto R, Diaz A (1999). Differential effects of respiratory syncytial virus and adenovirus on mononuclear cell cytokine responses. Am J Respir Crit Care Med.

[41] Asgari E, Le Friec G, Yamamoto H, Perucha E, Sacks SS, Kohl J, Cook HT, Kemper C (2013). C3a modulates IL-1beta secretion in human monocytes by regulating ATP efflux and subsequent NLRP3 inflammasome activation. Blood.

